# Iodine Injection of the Joints

**Published:** 1853-03

**Authors:** 


### SURGERY.

*Iodine Injection of the Joints.* By M. Velpeau.—Amongst the affections of the joints, effusion into them is a very common affection, but is only serious as a symptom of the disease which accompanies it. When it occurs as a serous interarticular effusion, without any marked material lesion, recent hydrops articuli is easily cured by rest, bleeding, and topical applications, such as solutions containing muriate of ammonia or chloride of sodium. When the effusion resists these means, M. Velpeau applies a large flying blister, repeated every fifteen days, and then uses frictions with mercurial or iodide of lead ointment (the latter being preferable to the iodide of potash,) aided by compression, the administration of calomel in small doses, and especially rest. There is another remedy now used—namely, the injection of tincture of iodine. M. Velpeau has only tried it twice this year; but from these two cases, it is plain that the injection thus used is neither very painful nor dangerous, and that when thus cured, the joint is not anchylosed. In one very bad case particularly, the injection was not more painful than when used for the cure of hydrocele, and succeeded where the other means referred to had failed. It is necessary that the treatment by iodine injection should be made more generally known, as it is not usually practised. The two points which deter surgeons from using it are, the fear of throwing an irritating fluid into a large joint, and of anchylosis taking place in case of success.

Now, both these dangers are imaginary. There is no previous incision, but a simple puncture made. Since 1839, M. Velpeau has used this plan twenty-five times, M. Bonnet perhaps as often, so that with cases of the same kind, related by Berard, and since by M. Jobert, Malgaigne, and other surgeons, there are more than one hundred cases of these joints having been punctured and treated by the iodine injection, and none of the patients have had any unfavorable symptom. The swelling, with slight redness, which appears after the operation, only shows that a natural process is going on, such as takes place in a hydrocele, and is resolved without the application of leeches, &c.

As to the danger of anchylosis, it is equally imaginary. M. Velpeau has seen these patients long after the operation, and in all the movements of the joints were preserved. It is, in fact, in these cases, as in hydrocele, the cure can be effected without the obliteration of the serous sac; or if adhesions do take place, they yield after a time, and the function of the joint is restored, so that this is no serious objection; and, as on the other hand, there is complete cure in one-half the cases, and very marked amelioration in the other, it is to be concluded that the iodine injection, under such circumstances, when as yet there is no induration, is suitable, and the more so, as its use does not prevent that of other accessory means of cure.—*Dublin Med. Press, from Presse Médicale de Belge.*

